# Characterization of Small RNAs Derived from tRNAs, rRNAs and snoRNAs and Their Response to Heat Stress in Wheat Seedlings

**DOI:** 10.1371/journal.pone.0150933

**Published:** 2016-03-10

**Authors:** Yu Wang, Hongxia Li, Qixin Sun, Yingyin Yao

**Affiliations:** 1 College of Agronomy, Northwest A&F University, Yangling, Shaanxi, China; 2 State Key Laboratory for Agrobiotechnology, Key Laboratory of Crop Heterosis and Utilization (MOE), Beijing Key Laboratory of Crop Genetic Improvement, China Agricultural University, Beijing, China; Institute of Crop Sciences, CHINA

## Abstract

Small RNAs (sRNAs) derived from non-coding RNAs (ncRNAs), such as tRNAs, rRNAs and snoRNAs, have been identified in various organisms. Several observations have indicated that cleavage of tRNAs and rRNAs is induced by various stresses. To clarify whether sRNAs in wheat derived from tRNAs (stRNAs), rRNAs (srRNAs) and snoRNAs (sdRNAs) are produced specifically in association with heat stress responses, we carried out a bioinformatic analysis of sRNA libraries from wheat seedlings and performed comparisons between control and high-temperature-treated samples to measure the differential abundance of stRNAs, srRNAs and sdRNAs. We found that the production of sRNAs from tRNAs, 5.8S rRNAs, and 28S rRNAs was more specific than that from 5S rRNAs and 18S rRNAs, and more than 95% of the stRNAs were processed asymmetrically from the 3’ or 5’ ends of mature tRNAs. We identified 333 stRNAs and 8,822 srRNAs that were responsive to heat stress. Moreover, the expression of stRNAs derived from tRNA-Val-CAC, tRNA-Thr-UGU, tRNA-Tyr-GUA and tRNA-Ser-UGA was not only up-regulated under heat stress but also induced by osmotic stress, suggesting that the increased cleavage of tRNAs might be a mechanism that developed in wheat seedlings to help them cope with adverse environmental conditions.

## Introduction

In eukaryotic organisms, small RNAs (sRNAs) of 18~30 nt have been found to play important roles in the regulation of gene expression. miRNAs and siRNAs are the most extensively studied sRNAs involved in RNAi in plants. Mature miRNAs are derived from hairpin precursors and are loaded onto Argonaute (AGO) proteins and guide the transcriptional or translational repression of target mRNAs. The miRBase (miRBase 21) website has 35,828 miRNA sequences deposited for all species. The development of next-generation sequencing (NGS) has rapidly increased the number of miRNAs identified in crops. New classifications of siRNAs have also been added to the known siRNA populations, such as trans-acting siRNA (ta-siRNA), natural antisense transcript siRNA (nat-siRNA), and repeat-associated siRNA (ra-siRNA) [1–3].

Increasing evidence has suggests that small RNAs are important regulatory components in plant development and stress responses [4, 5]. For example, manipulation of miR1848 and its targets in rice results in dwarf plants, erect leaves and semi-sterile pollen grains [6]. In barley, 44 miRNAs have been found to be differentially expressed in response to salinity stress, and miR160a, 166a, 167h and 5175a are up-regulated under heat stress [7, 8]. The expression of zma-miR169 and its targets exhibits diverse changes under drought as well as abscisic acid (ABA) and salt stress treatment in maize [9]. Recent studies have focued on the biological functions of siRNAs. For example, siRNAs derived from transposable elements (TEs) and repeats are broad regulators of the expression of nearby genes and affect the agricultural traits of rice [10]. In maize, 21 nt phased siRNA (phasiRNAs) have been shown to be spatiotemporally regulated during anther development, and ta-siRNA pathway mutants exhibit severe developmental defects [11, 12].

The discovery of novel sRNAs arising from non-coding RNAs, such as rRNAs, tRNAs, snoRNAs and snRNAs, has recently received significant attention. Small tRNA-derived fragments (tRFs) are classified into three types based on their relative location in tRNAs. 5’-tRFs are generated from the 5’ end of the tRNA through cleavage of the D loop by Dicer in in mammalian cells [13]. Dicer or other endonucleases (angiogenin and RNase A) are responsible for the production of 3’ tRFs, which originate from the 3’ extremity of mature tRNAs [14, 15]. 3’ U tRFs that match to the 3’ trailer of tRNA precursors, rather than mature tRNAs, are released by Dicer or RNase Z (ELAC2) activity [16–18]. It has been proposed that tRNAs must adopt alternative secondary structures to form a dsRNA that enables Dicer cleavage.

Several studies have shown that small tRFs are involved in the inhibition of protein synthesis and gene silencing. In the Archaeon *Haloferax volcanii*, a specific 26 nt-long 5’-tRF derived from tRNA-Val binds to the small ribosomal subunit, reducing protein synthesis by interfering with peptidyl transferase activity [19]. In humans, a conserved 5'-terminal oligoguanine (TOG) motif at the 5’ end of 5’-tRFs is required for translation inhibition [20, 21]. 3’-tRFs have been found to associate more effectively with AGO3 and AGO4 than with AGO1 or AGO2 and to have a moderate effect on reporter transgene silencing [17]. A 3’-tRF associated with AGO1 functions as an miRNA that represses replication protein A1 (RPA1) in mature B cells [14]. tRNA fragments have been shown to be selectively delivered in the phloem transport system as a small signaling molecule with the function of translational inhibition in pumpkins [22]. In Arabidopsis, both 5’-tRFs and 3’-tRFs can be loaded onto several AGO proteins and have been proposed to target mRNAs or to be involved in DNA chromatin modification [23]. tRFs play a role in gene silencing pathways, either by targeting mRNA sequences or by competing with the original small RNAs for loading onto the RISC complex [24].

In Arabidopsis and humans, small RNAs act as guide molecules in the double-strand break (DSB) repair signaling pathway [25]. In the filamentous fungus *Neurospora crassa*, qiRNA (QDE-2-interacting small RNA) has been found to be a new type of siRNA generated from the ribosomal DNA locus that mediates DSB repair in damaged repetitive rDNAs by inhibiting protein translation [26]. Similarly, srRNAs have been detected in high-throughput sequencing datasets of small RNAs from different species, and some srRNAs may be involved in RNAi-related mechanisms [27]. In human cells, 28S rRNA fragments function as small guide RNAs for tRNase Z^L^ to cleave target mRNAs [28]. In the Chinese sacred lotus, some large subunits (LSUs) are processed through the phasiRNA biogenesis pathway, which produces 21 nt phasiRNAs [29]. Two srRNAs detected in wheat root and shoot tissues are conserved in related plant species, suggesting that many srRNAs are not random degradation products [30].

Several reports have recently identified snoRNA-derived small RNAs (sdRNA) with miRNA-like functions through sRNA deep sequencing studies [31–33]. The secondary structure of the precursor RNA and Dicer cleavage are necessary for the processing of both sdRNAs and miRNAs. sdRNAs can also associate with AGO and participate in the suppression of gene expression [34–36]. In animals, sdRNAs are predominantly derived from the 3’ end of H/ACA snoRNAs and the 5’ end of C/D snoRNAs. [37, 38]. In Arabidopsis, sdRNAs with a 5’ U or 5’ A are preferentially associated with AGO7 [38]. Recently, functional analysis of sdRNAs revealed that they could affect the alternative splicing of mRNAs [39, 40].

RNA metabolism is an important component of translational repression and splice site alterations that occur in response to stress [41, 42]. Previous studies have shown that nutritional stress triggers the cleavage of specific tRNA isoacceptors in the anticodon loop, as an adaptation to starvation in *Tetrahymena thermophila* and *Trypanosoma cruzi* [43, 44]. In animals, various stresses promote angiogenin (ANG)-mediated tRNA cleavage, resulting in the inhibition of protein translation, induction of stress granule (SG) formation, preservation of cellular energy and protection of cells from apoptosis [45–48]. However, tRF accumulation is higher during oxidative stress compared with hypertonic stress [49]. In Arabidopsis and barley, tRFs have been shown to be involved in the phosphate deficiency and drought stress responses [23, 50, 51]. Small RNAs derived from tRNA intron regions have been predicted to be heat responsive and even transgenerationally transmitted [52].

Wheat (*Triticum aestivum*, AABBDD, 2n = 42) is the cereal with the third highest production worldwide. The optimum temperature for wheat growth is in the range of 17–23°C, and leaf photosynthesis declines above 37°C [53]. Hence, wheat is highly sensitive to heat stress and has developed subtle defense mechanisms to cope with elevated temperatures. A previous microarray study has shown that gene expression related to RNA metabolism and ribosomal proteins is affected by heat stress [54]. Recently, a draft of the wheat genome has been reported, providing a reference sequence for small RNA analysis [55, 56]. Previous studies have discovered numerous miRNAs in various tissues and under different conditions by constructing small RNA libraries [57–64]. Moreover, 150 known and 100 novel miRNAs have been identified to be differentially expressed between wild-type and a high-yield wheat strain [65]. Heat-responsive miRNAs in wheat have been intensively explored in a recent study using the reference genomes of wheat and related plant species [66]. However, more than 80% of the wheat genome consists of repeated sequences [67]. Transposable element associated siRNAs (TE-siRNAs) in wheat sRNA libraries have been found to function in grain development and to contribute to speciation through RNA-directed DNA methylation (RdDM) [68–70]. In addition to miRNAs and siRNAs, wheat sRNA libraries contain sequences derived from ncRNAs. Given that sRNAs derived from non-coding RNAs have been proven to play a vital role in the process of abiotic stress tolerance in other species, we speculated that wheat sRNAs might respond to heat stress.

In this study, we reanalyzed the sRNA libraries of wheat seedlings grown under normal and high-temperature conditions to characterize sRNAs derived from tRNAs, rRNAs and snoRNAs. We found that stRNAs, srRNAs and sdRNAs were not random by-products of non-coding RNA degradation, according to their size, positional distribution and the nucleotide composition of cleavage sites. Furthermore, we identified stRNAs associated with heat and osmotic stresses.

## Results

### Identification of Non-Coding Small RNAs in Wheat Seedlings

**Fig 1:**
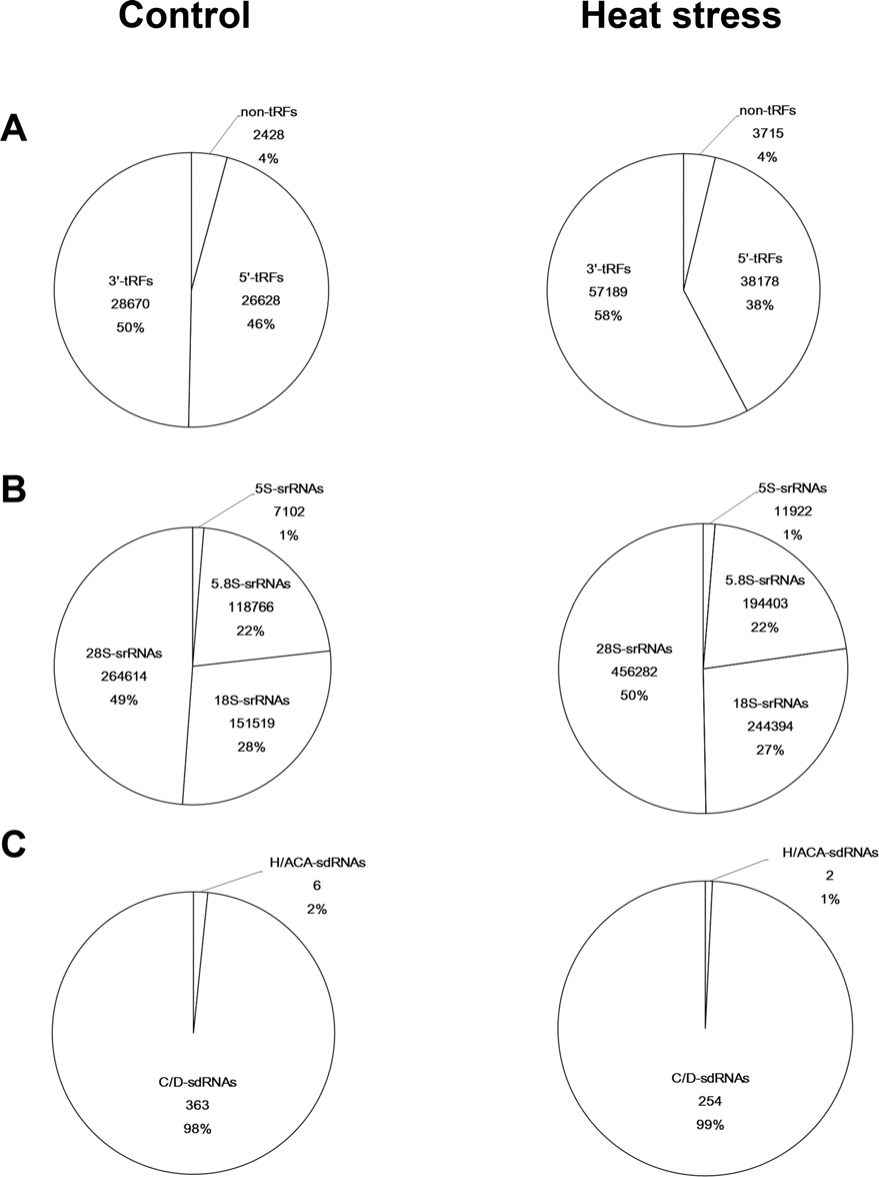
Classification of stRNAs, srRNAs and sdRNAs. (A) stRNAs were divided into non-tRFs and tRFs according to their positions in the tRNAs. (B) srRNAs were classified according to the rRNA species, including 5S rRNA, 5.8s rRNA, 18S rRNA and 28S rRNA. (C) sdRNAs were divided into two types on the basis of their snoRNA classifications.

In a previous study, our analysis focused mainly on miRNAs in sRNA libraries constructed from wheat seedlings of the heat-tolerant cultivar TAM107 that were grow under either normal conditions or under heat stress for 1 hr, which yielded 7,852,141 pre-processed total reads of 2,467,900 unique small RNAs [71]. In the present study, the sRNAs profiles associated with tRNAs, rRNAs and snoRNAs were characterized using these sRNA libraries to investigate the features of these ncRNA-derived sRNAs and their relationships with the heat stress response. After extracting predicted miRNAs that occupied ~0.5% of the combined reads, a strikingly high percentage of ncRNA-derived sRNAs were identified by mapping the remaining sRNAs onto the tRNA, rRNA and snoRNA sequences in the Rfam database, the wheat A and D genomes and the IWGSC database (http://ensemblgenomes.org/), and multiple matching loci were allowed. Moreover, the total percentage of ncRNA-derived sRNAs in the heat stress dataset (25%) was even higher than in the control libraries (15.3%) (Table 1). The most abundant class of ncRNA-derived sRNAs in our libraries was generated from rRNAs, and the vast majority (50%) of srRNAs were aligned to eight 28S rRNAs, whereas those generated from snoRNAs were the least prevalent (0.01%) (Fig 1, Table 1). stRNAs represented the second most abundant class of ncRNA-derived sRNAs, but the abundance/unique numbers values (~300) were much higher than those of srRNAs and miRNAs, suggesting that stRNAs were more specifically produced. The large number of sRNAs derived from tRNAs, rRNAs and snoRNAs and their variation between the two libraries indicated that ncRNA-derived sRNAs might play an important role in the heat stress response, in addition to miRNAs.

**Table 1:** Summary of read mapping statistics.

Classification of small RNAs	Number of unique small RNAs		Reads of small RNAs	
	Control (%)	Heat stress (%)	control (%)	Heat stress (%)
miRNA	814 (0.06%)	697 (0.05%)	23762 (0.61%)	15659 (0.39%)
stRNA (450)*	1656 (0.12%)	2073 (0.16%)	57725 (1.49%)	99080 (2.48%)
srRNA (2130)*	37432 (2.65%)	46205 (3.63%)	537351 (13.90%)	898869 (22.54%)
sdRNA (197)*	259 (0.02%)	175 (0.01%)	369 (0.01%)	256 (0.01%)
others	1370671 (97.15%)	1222345 (96.13%)	3245604 (83.98%)	2973466 (74.57%)
All	1410832 (100%)	1271495 (100%)	3864811 (100%)	3987330 (100%)

* indicates the number of ncRNAs mapped to sRNAs.

### Distribution of sRNAs in tRNAs, rRNAs and snoRNAs

**Fig 2:**
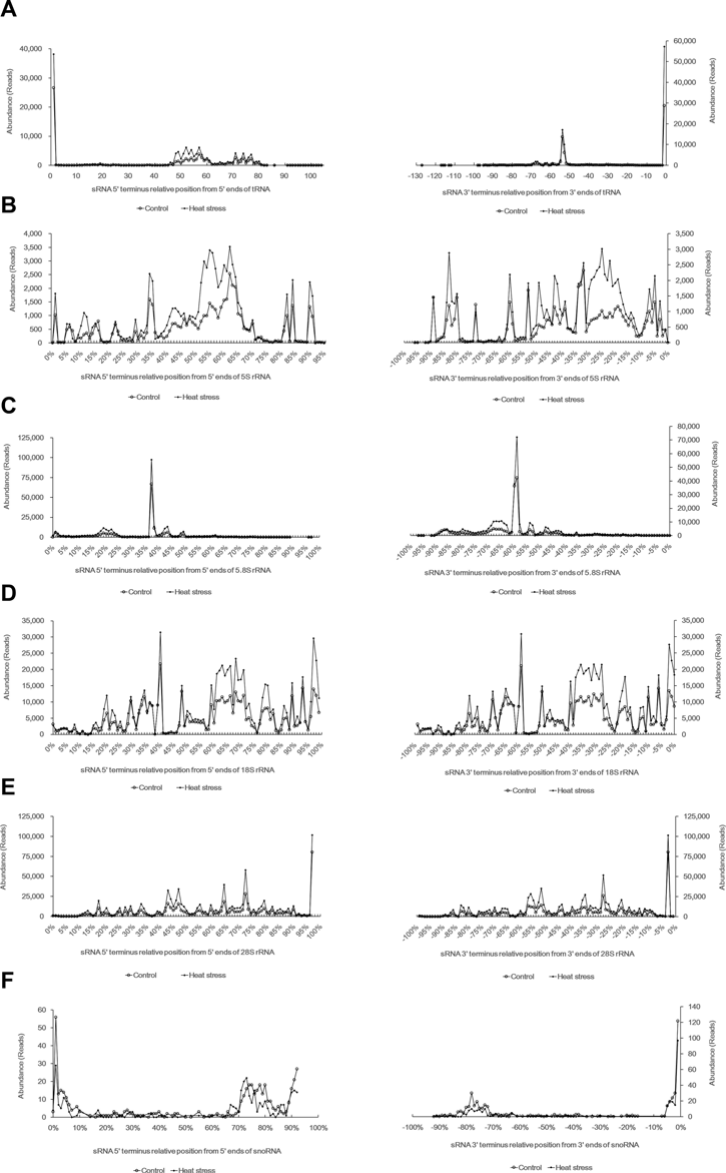
Abundance of sRNAs mapped to tRNAs, rRNAs and snoRNAs. The 3’ and 5’ ends of the sRNAs were plotted onto tRNAs, rRNAs, snoRNAs. The left panels show the positions of the sRNA 5’ ends, and the right panels show the relative postions of the sRNA 3’ ends. (A) Abundance of sRNAs mapped to tRNAs. The first nucleotide of the tRNAs was set as 1. Two peaks were formed at both the 5’ and 3’ termini of the tRNAs (B) Abundance of sRNAs mapped to 5S rRNAs. (C) Abundance of sRNAs mapped to 5.8S rRNAs. The sRNA reads are clustered at the 40% position of rRNAs. (D) Abundance of sRNAs mapped to 18S rRNAs. (E) Abundance of sRNAs mapped to 28S rRNAs. Only one peak was produced at the 3’ terminus of 28S rRNA. (F) Abundance of sRNAs mapped to snoRNAs. The sequences of the full-length rRNAs and snoRNAs were divided into 100 fractions.

In most eukaryotes, a ‘CCA’ oligonucleotide added to the 3’ end of the trimmed tRNA transcript facilitates ribosome interactions [72]. To characterize the sRNAs generated by mature tRNAs, a ‘CCA’ motif was also added to the 3’ end of the tRNAs before alignment. We found two strong peaks at both the 5’ and 3’ ends of the mature tRNAs, where more than 96% of the reads from the two libraries were mapped (Fig 2A). Traditionally, sRNAs that precisely map to the 5’ or 3’ end of mature or tRNAs are defined as 5’-tRFs or 3’-tRFs. Among the 3’-stRNAs (3’-tRF) reads, 99% contained an intact ‘CCA’ motif, which was cleaved from the TΨC arms (S1 Table). This finding supported the notion that wheat stRNAs are predominantly generated from both ends of mature tRNAs, rather than from nascent tRNA transcripts [23]. However, sRNAs originating from the central region of tRNAs have also been identified in human cells and plants [23, 73, 74]. For example, we found that all sRNAs derived from tRNA-Gln-CTG could be mapped to the central region, as reported in human cells (S1 Table) [75]. Hence, sRNAs derived from the tRNA central region (non-tRFs) were also included in our analysis, in spite of their low abundance. Notably, although 35% (905/2587) of the stRNAs mapped to more than one site of the 450 tRNA sequences, no stRNAs fell under more than one classification. Approximately 70% of the non-tRFs spanned the anticodon loop, suggesting that the anticodon loop might be another common source region of stRNA generation. As shown in Fig 1A, an approximately 10% difference in the stRNA composition was observed between the control and treated samples.

Given that stRNAs clustered in the double-stranded regions of the secondary structure of the ncRNA sequences, we speculated that base pairing was necessary to generate the sRNAs that resembled miRNAs. It has been suggested that tRFs may be generated asymmetrically from the 5’ and 3’ ends of tRNAs in mammals due to the acceptor stem duplex in the cloverleaf structure [76]. To explore this possibility in wheat, we compared the abundance of 5’-tRFs to that of 3’-tRFs in each of the identified unique tRNA sequences. Among the 450 tRNA sequences that matched tRFs, 91 (20%) tRNAs mapped to both 3’-tRFs and 5’-tRFs (S1 Table). However, for 90% (82/91) of those tRNAs, two-thirds of the tRFs were from either the 3’ or 5’ region, indicating that the cleavage of the tRNAs in the wheat seedlings also was also asymmetrical.

We identified 55,336 distinct sRNAs corresponding to 537,351 and 898,869 reads in the two wheat seedling libraries that were mapped to rRNAs. To investigate the distribution of srRNAs along the rRNAs, we divided each rRNA sequence into 100 blocks from the 5’ to the 3’ ends, and we summed the reads for each block, which was similar to the method described by Wang [77]. An enrichment of the origin of srRNAs was found in 40% of the 5.8S rRNA and the last 3% of 28S rRNAs, whereas the srRNAs mapped to the 18S rRNA and 5S rRNAs at random positions, indicating that the srRNAs were processed from specific regions of the 5.8S and 28S rRNAs (Fig 2B, 2C, 2D and 2E).

We identified 397 distinct sRNAs that originating from 197 snoRNA sequences in the two libraries. Although each sdRNA represented a low abundance, 90% of the reads exclusively mapped to the 3’ and 5’ ends of snoRNAs where hairpin structures formed (Fig 2F). It has been postulated that miRNAs evolved from H/ACA box snoRNAs [35]. We attempted to align the miRNAs in these two libraries with snoRNAs; however, no sequence similarity was detected between the predicted miRNAs and snoRNAs.

### Length Distribution and Nucleotide Composition at Cleavage Sites

**Fig 3:**
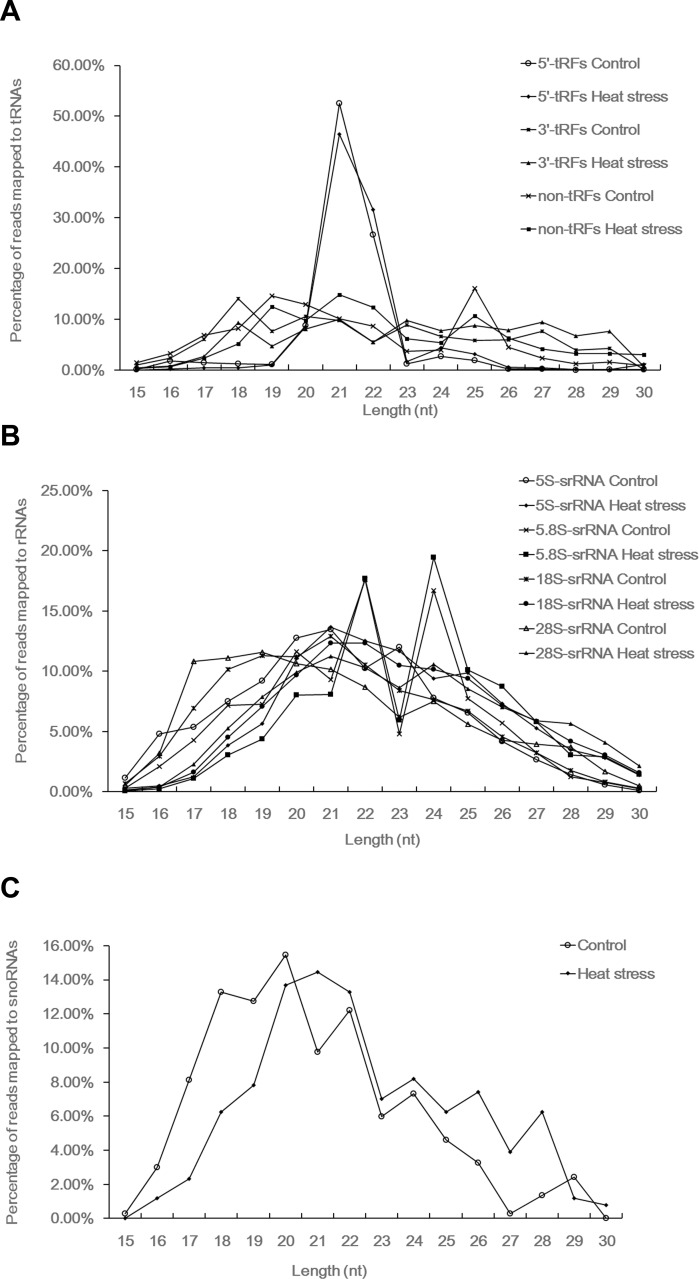
The size distribution of sRNAs derived from tRNAs, rRNAs and snoRNAs. (A) Size distribution of 5’-tRFs, 3’-tRFs and non-tRFs; (B) size ditribution of 5S-srRNAs, 5.8S-srRNAs, 18S-srRNAs and 28S-srRNAs; (C) size distribution of sdRNAs.

The length distribution and nucleotide preference at cleavage sites of the sRNAs may be suggestive of their biogenesis and regulation mechanisms, similar to miRNAs. The majority of the 5’-tRFs present in the wheat seedlings were 21 nt in length, followed by 22 nt signatures. The greatest accumulation of non-tRFs by length occurred at 19–22 nt, with a small peak at 25 nt. In contrast, the 3’-tRFs occupied a broad size range of 18–29 nt (Fig 3A). The length of the srRNAs from the 5S, 18S and 28S RNAs exhibited similar characteristics, showing a normal distribution pattern in the 15–30 nt size range; however, the length distribution of the 5.8S-derived srRNAs was bimodal, at 22 nt and 24 nt (Fig 3B). The lengths of the sdRNAs were concentrated approximately between 18 and 22 nt (Fig 3C).

**Fig 4:**
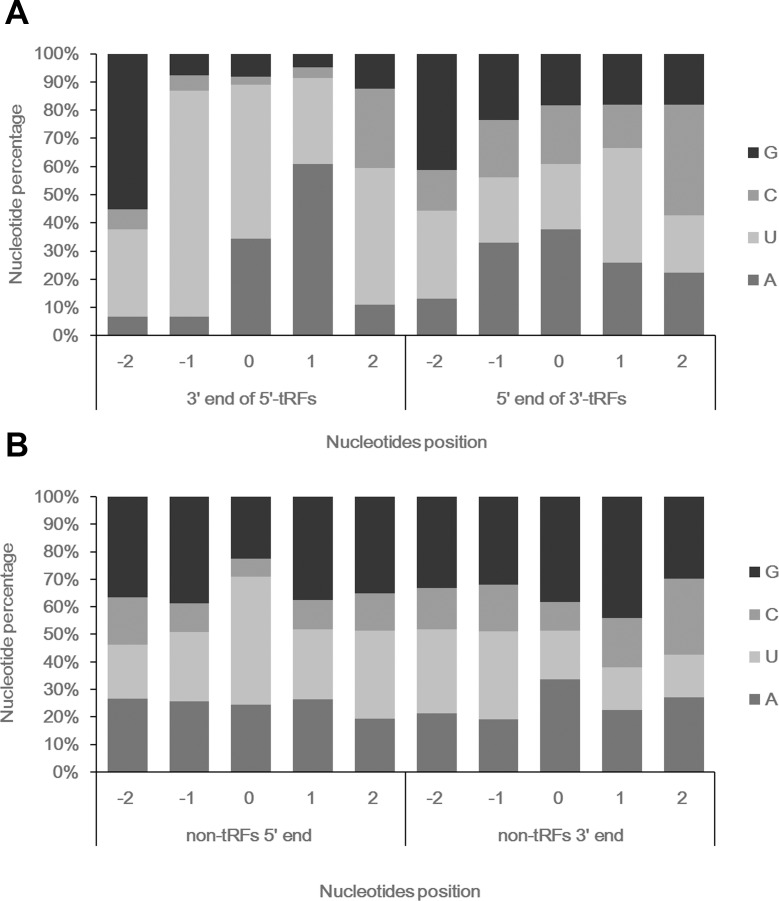
Nucleotide composition around cleavage sites. The numbers on the X-axis represent the relative position from the terminal nucleotides of the sRNAs, which was assigned as ‘0’. -1 and -2 are the first and second nucleotides upstream of the cleavage site, while +1 and +2 are the first and second nucleotides downstream of cleavage site. (A) Nucleotide composition around the 5’-tRFs and 3’-tRFs cleavage sites. (B) Nucleotide composition around the cleavage sites at both ends of the non-tRFs.

The nucleotide composition of tRFs around cleavage sites was examined using the combined reads from the two libraries. Considering that over 96% of the stRNAs were classified as tRFs, we only analyzed the nucleotide composition around the 5’ ends of the 3’-tRFs and the 3’ ends of the 5’-tRFs. Cleavage at the 5’ ends of the 3’-tRFs displayed a more obvious nucleotide preference than that at the 3’ ends of the 5’-tRFs (Fig 4A). U was enriched at all positions around the 3’ ends of the 5’-tRFs, and cleavage tended to occur at a U/A between U (-1) and U/A (+1), suggesting that the processing of the 5’-tRFs was dependent on a certain motif around the cleavage site. However, U was preferred at only the -2 and +1 positions of the 3’-tRFs 5’ ends. The 5’ ends of the central tRFs also showed preferential cleavage at U, whereas other positions showed no nucleotide preference (Fig 4B). The enrichment of U in the vicinity of the cleavage sites and the nonrandom size distribution of the tRFs supported the notion that they were generated from the specific cleavage of tRNA molecules and may be tightly regulated in wheat seedlings.

### tRF Generation Is Associated with Certain Amino Acid Types

tRFs were also non-uniformly derived from all of the tRNA families. The abundance of the tRFs mapped to different types of tRNAs revealed that four types dominated: Val, Ser, Thr and Tyr, which belong to the hydrophobic and polar amino acids (S1 Table). In fact, more than 70% of the tRF reads matched tRNAs that carried those two amino acids types, suggesting that the nature of the tRNA may be one of the factors involved in tRF generation. The abundance of tRFs from the shared tRNA types carrying various anticodon isoacceptors appeared to be inconsistent. For example, tRNA-Val has four isoacceptors, among which tRNA-Val -AAC and tRNA-Val-CAC were present at significantly higher tRF levels than the others. These findings demonstrated that anticodon isoacceptors are another factor involved in the tRF processing, in addition to the nature of amino acids.

### Response of stRNAs, srRNAs and sdRNAs to Heat Stress

**Fig 5:**
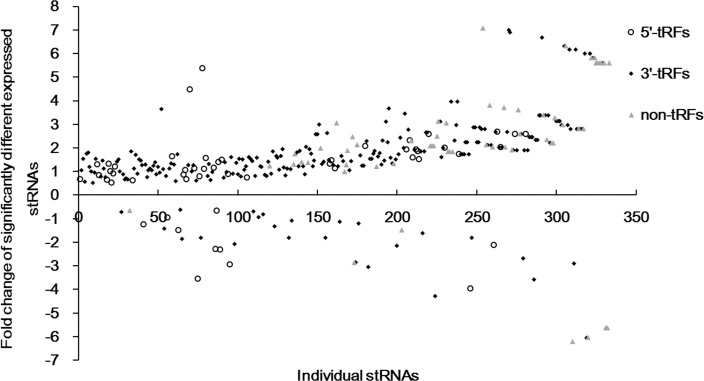
Fold change of the significantly differentially expressed stRNAs. The stRNAs were sorted based on their combined abundance in the two libraries, from high to low.

To test whether the tRNA-derived sRNAs found in wheat seedling leaves might be involved in the response to heat stress, we used the Bioconductor package edgeR to identify sRNAs showing a statistically significant difference in relative abundance between the two libraries (P-value = 0.05, bcv = 0.1). Among the 2,587 identified stRNAs, the abundance of the 292 stRNAs was increased, whereas that of 41 tRNAs was decreased significantly under high temperature (Table 2). Most of these dramatically altered stRNAs (67%) were classified as 3’-tRFs, which suggested that heat stress resulted in increased cleavage of the 3’ ends of the tRNAs. As shown in Fig 5, the expression of 90% of the stRNAs with high abundance (>100) was significantly up-regulated by approximately two-fold. Some of the tRFs from the same amino acid isotype displayed diverse expression patterns, such as Ta-stRNA0120d from tRNA-Met-CAU, which exhibited lower abundance during heats stress, however, Ta-stRNA0031d, which is derived from the same isoacceptor, showed increased accumulation (S2 Table). Additionally, the total number of sRNAs spawned by some of these tRNAs, which reflected the propensity toward cleavage of tRNA molecules, was found to be significantly different between the two libraries. As expected, the cleavage of 130 (29%) tRNAs dramatically increased in response to heat stress, whereas that of 13 tRNAs decreased (S1 Table). The instability of tRNAs under high temperature might account for the increased tRF levels.

**Table 2:** The number and abundance of differentially expressed sRNAs derived from tRNAs, rRNAs and snoRNAs.

			Up			Down	
Classification of sRNAs		sRNA number	Control	Heat stress	sRNA number	Control	Heat stress
stRNA	5'-tRF	42	10832	20203	10	1223	387
	3'-tRF	200	20516	48698	24	1882	830
	non-tRF	50	251	1024	7	395	216
srRNA	5S-srRNA	148	2072	5978	17	664	155
	5.8S-srRNA	438	53340	105090	58	14148	6767
	18S-srRNA	2409	53913	142833	496	23581	9005
	28S-srRNA	4733	110928	293112	523	36105	10962
sdRNA	CD-sdRNA	1	6	6	0	0	0
	H/ACA-sdRNA	0	0	0	0	0	0

**Fig 6:**
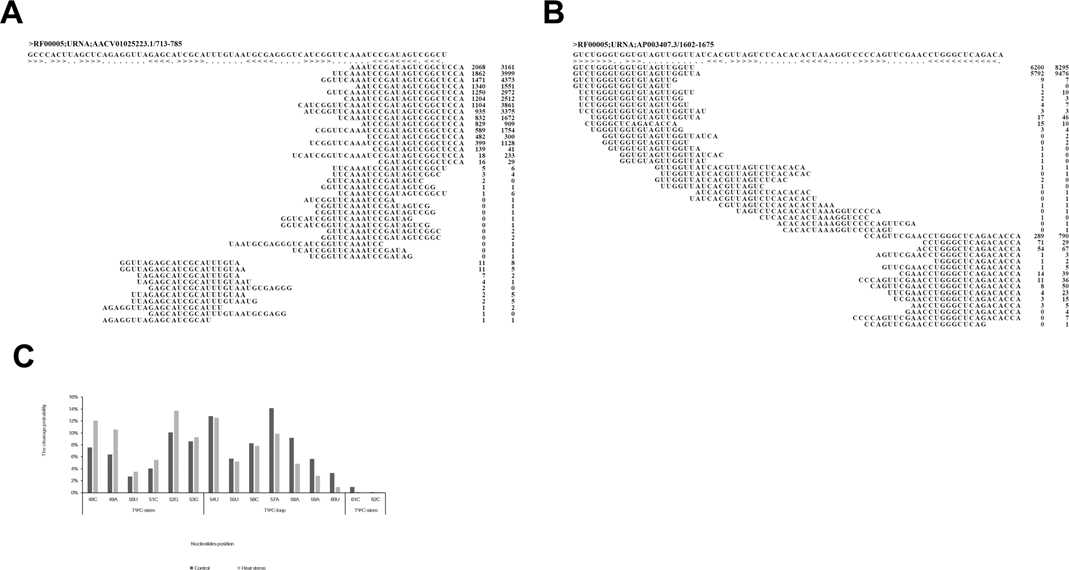
Examples of two tRNAs that aligned with sRNAs. The numbers following the sRNA sequence are the abundance values in the two sRNA libraries. The first number represents the reads in the control library, and the second number represents the reads in the heat stress library. (A) tRNA-Thr-UGU. (B) tRNA-Val-CAC. (C) The cleavage probability of a given site on the TΨC-arm of tRNA-Thr-UGU in the two libraries.

The most frequently sequenced tRFs that were induced by heat stress came from tRNA-Val, followed by tRNA-Thr, which constituted approximately one-third of the sRNA reads that mapped to tRNAs. We analyzed tRNA-Thr-UGU and tRNA-Val-CAC in detail to characterize their tRNA cleavage properties in response to heat stress (Fig 6). Approximately 99% of the reads aligned to the 3’ end of the tRNA-Thr-UGU sequence and were cleaved on the TΨC arm (Fig 6A). Processing in the TΨC loop was reduced after heat stress, whereas in the TΨC stem, processing was slightly increased, at A57 and A49 for example (Fig 6C), suggesting that the TΨC stem is less stable than the TΨC loop under higher temperatures. In contrast, tRNA-Val-CAC showed a preference for producing sRNAs from the 5’ end within the D-loop, ~95% of the time at T21 and A22, in the two libraries (Fig 6B).

**Fig 7:**
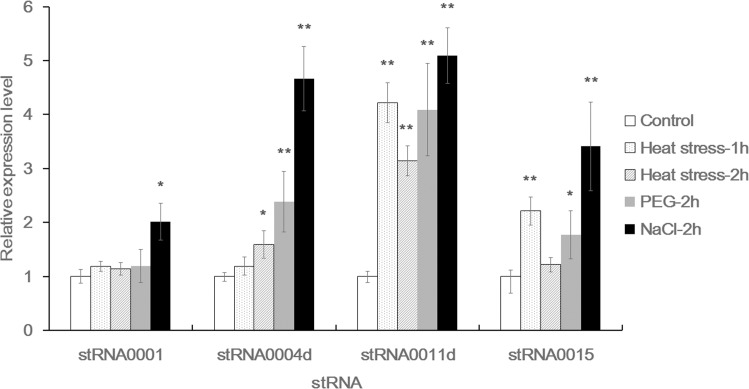
tRFs detection via real-time RT-PCR. The levels four tRFs were estimated through real-time RT-PCR in the seedlings. The expression levels were normalized to the control samples. The asterisks mean that the values were statistically different from the controls, as determined by Student’s *t*-test (**, P < 0.01; *, P < 0.05).

We used real-time RT-PCR to detect the expression patterns of four tRFs derived from tRNA-Val-CAC, tRNA-Thr-UGU, tRNA-Tyr-GUA and tRNA-Ser-UGA in TAM107 under abiotic stress conditions, such as heat, drought and salt treatments. The results indicated that the four tRFs were all induced by NaCl stress (Fig 7). The expression levels of stRNA0001 and stRNA0004d remained unchanged after heat stress, which differed from the deep sequencing results. However, stRNA0011d and stRNA0015 were up-regulated by high-temperature and dehydration stress, suggesting that the expressions of some tRFs is responsive to different abiotic stresses.

We observed that the levels of 14% (7728/55336) of the srRNA increased significantly, whereas those of 2% (1094/55336) of the srRNAs decreased during exposure to heat stress (S3 Table). However, the abundance/unique number values obtained for 5.8S were even higher than those of the 3’-tRFs (Table 2), which indicates that stress-induced cleavage of the RNAs was not restricted to tRNAs but also involved other RNA species, such as 5.8S rRNAs. Intriguingly, the length of the srRNAs was also sensitive to heat stress, because the abundance of longer srRNAs (21–30 nt) tended to increase, whereas that of shorter srRNAs (15–20 nt) decreased significantly after heat stress (S3 Table).

## Discussion

### sRNAs Are Specifically Produced from tRNAs, rRNAs and snoRNAs

The identification of ncRNA-derived sRNAs sheds light on RNA metabolism related to development and stress responses. A large number of ncRNA-derived sRNAs have been recently found in Arabidopsis, barley, rice, Brassica and Chinese sacred lotus. Our findings provide evidence of the existence of tRNA-, rRNA- and snoRNA-derived sRNAs in wheat, which were filtered out as sequencing noise during miRNA identification. We referred to the Rfam database, because the available wheat genome was imperfect. Nevertheless, 2,587 stRNAs, 55,336 srRNAs and 397 sdRNAs were obtained from the two wheat seedling sRNA libraries, and their proportions increased when the temperature rose abruptly, which suggested that the production of ncRNA-derived sRNAs was induced by heat stress.

In the B lymphoma BCP1 cell line, sRNAs are preferentially produced at the 5’ and 3’ ends of tRNAs, snoRNAs, rRNAs and snRNAs [76]. A similar phenomenon was observed in sRNAs derived from tRNAs and snoRNAs in wheat seedlings. However, srRNAs were found to largely be derived from specific regions of 5.8S and 28S rRNAs, rather than the terminal region, where regions of interaction have been reported [78]. 5.8S rRNA and 28S rRNA are components of the large subunit of the eukaryotic ribosome that can be processed into phasiRNAs in the Chinese sacred lotus. Moreover, 5.8S-srRNAs that were differentially expressed in response to heat stress were more specifically produced than other classes of srRNAs, according to the obtained abundance/unique number values (Table 2). The positional distribution of the sRNAs and their high abundance provide evidence that sRNAs derived from tRNAs, rRNAs and snoRNAs are selectively processed, rather than simply representing by-products of deep sequencing in wheat and other species.

### Both 5’-tRFs and 3’-tRFs May Be Generated from tRNAs

In plants, 5’-tRFs have been intensively studied because of their high abundance. In Arabidopsis, 5’-tRFs with a length of 19 nt formed the most abundant class associated with AGO, and 80% of the tRFs are derived from tRNA-Gly-UCC [23, 79]. In rice leaves, the length of most 5’-tRFs is approximately 25 nt, and the most abundant tRFs come from tRNA-Ala-AGC [78]. In wheat, the predominant 5’-tRF sizes were 21 nt and 22 nt, and 5’-tRFs originating from tRNA-Val-CAC were the most abundant derivatives. It appeared that tRNA cleavage is dependent on tRNA species and their anticodon usage. Additionally, the nucleotide composition around the 5’-tRF cleavage sites suggested that some specific endonucleases might be responsible for the production of 5’-tRFs. In humans, the cleavage of 5’ tRNA ends is Dicer dependent, but the biogenesis of 5’-tRFs in wheat remains to be investigated. It has been demonstrated that 5’-tRFs with a 5’ TOG motif and a stem-loop act as translation inhibitors [15]. Among the differentially expressed stRNAs, we identified eight stRNAs with a 5’ TOG structure. These results suggested that 5’-tRFs are induced by heat stress and might be involved in translational regulation.

In fact, more than half of the heat stress-responsive stRNAs were found to be cleaved from the 3’ ends of tRNAs with a ‘CCA’ motif. The 3’ end of tRNA-Tyr-UGU gave rise to the most abundant tRFs without a size preference. It has been proposed that the selective stabilization of 3’-tRFs and 5’-tRFs is responsible for the asymmetric processing of tRNAs, which resemble the ‘mature’ and ‘star’ strands of precursor miRNAs [80]. We observed that 3’-tRFs were the dominant products of tRNA-His-GUG, which yields 5’-tRFs in barley [51]. These comparisons suggest that the terminal and asymmetric generation of stRNAs is conserved between mammals and plants; however, the selection of the tRNA cleavage position occurs in a species- specific manner.

### stRNAs and srRNAs Are Induced by Heat Stress

Heat stress can induce inhibition of global protein synthesis and alter free amino acids levels. We performed comparisons between control and high-temperature-treated samples to measure the differences in the abundance of stRNAs associated with heat stress. This analysis revealed that heat stress increases the circulating levels of small RNAs derived from specific tRNA isoacceptors, such as tRNA-Val-CAC. In human cells, tRNA cleavage is greater after oxidative stress compared with hypertonic stress [49]. We observed that a tRF derived from tRNA-Tyr-GUA was induced by heat, dehydration and NaCl stress, and its expression was greatly increased under NaCl stress.

It has been proposed that srRNAs might be generated through rRNA degradation to eliminate defective rRNA molecules, or from fragmentary rRNA transcripts with a specific secondary structure in mammals. Nevertheless, srRNAs associate with AGO and are involved in energy metabolism [27]. We demonstrated that srRNA levels are correlated with the heat stress response according to the expression profiles of srRNAs in wheat seedlings.

## Materials and Methods

### Plant materials and stress treatments

The heat-tolerant genotype 'TAM107' was used in this study. Seeds were surface-sterilized in 1% sodium hypochlorite for 15 min, then rinsed in distilled water and soaked in the dark overnight at room temperature. The germinated seeds were transferred into 25 cm-diameter pots (25 seedlings per pot) containing vermiculite. After one week, three pots regarded as three biological replicates were subjected to heat (40°C) treatment for 1 or 2 hours, and three pots were subjected to dehydration treatment (25% PEG6000 for 2h), and another three pots were treated with NaCl (200 mM NaCl for 2 h). Leaves were collected at 0, 1 and 2 hours after the heat treatment. At the end of each treatment, the leaves of the 10 seedlings in the center of pots were immediately frozen in liquid nitrogen and then stored at -80°C for further use.

### Real-Time RT-PCR Analysis of tRFs

Small RNAs were isolatied with the miRcute miRNA kit (Tiangen Inc. Beijing. China, DP501) and the first-strand cDNA was synthesized using the miRcute miRNA First Strand cDNA Synthesis kit (Tiangen Inc. Beijing. China, KR201) following the manufacturer’s instructions. Real-time PCR analysis was conducted using BioRad CFX 96 with SYBR Green Premix (Tiangen Inc. Beijing. China, FP401). The wheat gene U6 was used as the internal reference gene. The mean of three technical replicates was taken as the expression level of one biological replicate. Student’s *t*-test was used to analyze the expression data, and *P*-values of < 0.01 or 0.05 were considered statistically significant. The tRF-specific forward primers are listed in S4 Table. The specificity of the PCR assays for tRFs was detected using gel electrophoresis (S1 Fig).

### Small RNA Sequences Processing

Raw small RNA sequences that have been deposited in NCBI (accession number GSE27339) were used. All of the sequencing data were trimmed of 3' and 5' adapter sequences for further analysis.

The raw data were processed as depicted in S2 Fig.

1. The sRNA sequences were subjected to miRNA prediction based on the wheat genome deposited at the IWGSC (http://www.wheatgenome.org/), and the predicted miRNA sequences were discarded.
2. The remaining sRNA sequences were used for alignment with the tRNAs (‘CCA’ added), rRNAs and snoRNAs annotated in the wheat genome (IWGSC) and in wheat genomes A and D using Bowtie software. The perfectly matched tRNAs, rRNAs and snoRNAs were retained for further use.
3. The sRNAs that were not matched with ncRNAs in the wheat genome were mapped to tRNAs (‘CCA’ added), rRNAs and snoRNAs in the Rfam database (http://rfam.sanger.ac.uk/). The perfectly matched tRNAs, rRNAs and snoRNAs were subjected to BLAST searches against the wheat genome (IWGSC) and wheat genomes A and D at an 80% similarity cutoff, using the BLAST score and required identity to confirm that the tRNAs, rRNAs and snoRNAs screened from the Rfam database were homologous to those of wheat.
4. The tRNAs, rRNAs and snoRNAs obtained from steps 2) and 3) were clustered in each category to eliminate redundant ncRNAs. The sequences showing more than 90% identity were considered to represent a single ncRNA sequence.
5. The sRNAs that mapped to the remaining tRNA, rRNA and snoRNA sequences were analyzed to determine the read count for each ncRNA sequence.
6. The sRNA reads were further analyzed with the Bioconductor package edgeR to detect the differences between the samples before and after heat stress.

## Supporting Information

S1 Fig Detection of the specificity of real-time RT-PCR products via 2% agarose gel electrophoresis. The tRFs PCR products were 40–60 bp in length, and a negative control without template added was utilized.
(TIF)

S2 Fig Pipeline of small RNA sequences processing. (TIF)

S1 Table Details of read matching to the tRNAs and tRNA expression. (XLSX)

S2 Table stRNAs that were significantly differentially expressed after heat stress identified in wheat seedling leaves. The underlined stRNAs contained a TOG motif.
(XLSX)

S3 Table The srRNAs that were significantly differentially expressed after heat stress identified in the wheat seedling leaves. (XLSX)

S4 Table The primer sequences used for real-time RT-PCR. (XLSX)
