# Supplementary figures and images for: Characterization of Small RNAs Derived from tRNAs, rRNAs and snoRNAs and Their Response to Heat Stress in Wheat Seedlings
Source: PLoS One. 2016 Mar 10;11(3):e0150933. doi: 10.1371/journal.pone.0150933 (PMC4786338; doi:10.1371/journal.pone.0150933)

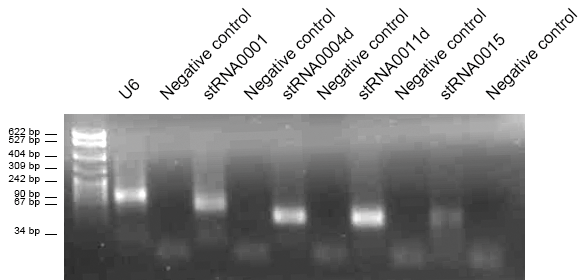

Supplement: S1 Fig — The tRFs PCR products were 40–60 bp in length, and a negative control without template added was utilized. (TIF) [file pone.0150933.s001.tif]

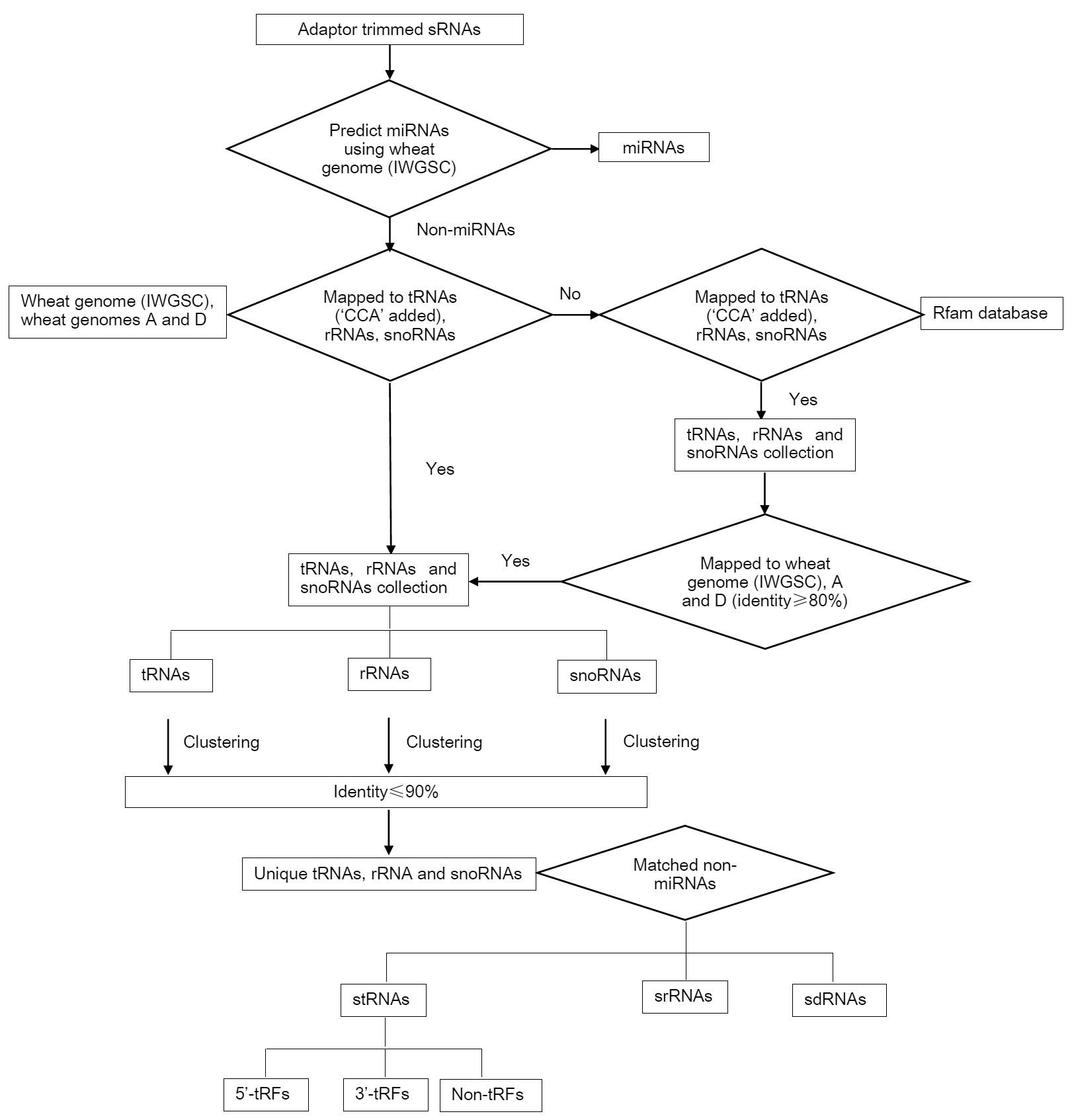

Supplement: S2 Fig — (TIF) [file pone.0150933.s002.tif]
